# Chitosan Nanoparticle Based Mucosal Vaccines Delivered Against Infectious Diseases of Poultry and Pigs

**DOI:** 10.3389/fbioe.2020.558349

**Published:** 2020-11-13

**Authors:** Sankar Renu, Gourapura J. Renukaradhya

**Affiliations:** ^1^Food Animal Health Research Program, Ohio Agricultural Research and Development Center, Wooster, OH, United States; ^2^Department of Veterinary Preventive Medicine, College of Veterinary Medicine, The Ohio State University, Columbus, OH, United States

**Keywords:** chitosan nanoparticle, infectious disease, mucosal vaccine delivery, poultry, pig, immune response, protection

## Abstract

Infectious disease of poultry and pig are major threat to health and cause severe economic loss to the food industry and a global food safety issue. Poultry and pig act as a mixing vessel of zoonotic transmission of disease to humans. Effective mucosal vaccines used in animals could reduce the impact of diseases in food animals. Chitosan is a biocompatible polymer, and its positive charge makes it a natural mucoadhesive agent. Therefore, since last one-decade chitosan derived nanoparticles (CS NPs) have been in use widely to deliver vaccine antigens in animals through mucosal route. Primary route of entry of most infectious disease pathogen is through oral and nasal routes, and the CS NPs based vaccines delivered through that routes enhance the immunogenicity of encapsulated vaccine antigens by targeting the cargo to mucosal microfold cells, dendritic cells and macrophages. Resulting in induction of robust secretory and systemic antibodies and/or cell mediated immune response which provides protection against infections. To date, CS NPs is being widely used for mucosal vaccine delivery in poultry and pigs to control bacterial and viral infections, and tested in several preclinical trials for vaccine delivery in humans. In this review, we highlighted the progress so far made in using CS NPs as a vehicle for mucosal vaccine delivery against infectious and zoonotic diseases of poultry and pigs. Discussed about the need of CS NPs modifications, CS NPs based vaccines induced immune responses and its role in protection, and challenges in vaccination and future directions.

## Introduction

Traditionally vaccination is the preferred and effective approach to control and prevent infectious diseases (Guo et al., 2019). The innovative vaccine preparation provides specific adaptive immunity and prevent many pathogenic infections (Yu et al., 2019). Several antigenic forms are used as a vaccine candidate such as live attenuated and inactivated pathogens, subunit protein, and recombinant protein/gene of pathogens (Guo et al., 2019). Delivery of vaccine antigens in their native form have several limitations such as poorly immunogenic, easily degradable in the body, immune tolerance, and toxic (Fowler et al., 2012; Sridhar et al., 2015). Hence, several approaches are used to overcome such problems by using appropriate vaccine delivery system and specifically targeting vaccine to antigen presenting cells, use of adjuvants and suitable route of administration (Yu et al., 2019). For most infectious disease inactivated pathogen is used as a vaccine, and the licensed inactivated pathogen vaccines are delivered through parenteral route. The limitations of injectable vaccines include expensive, low compliance and importantly lack ability to induce mucosal immunity (Pawar and Jaganathan, 2016). Majority of bacterial and viral pathogens use mucosal surfaces such as nasal, oropharyngeal, respiratory, gastrointestinal and urinogenital tract as their primary route of entry into the body, thus vaccines administrated through mucosal routes is likely a suitable strategy to trigger protective immune response (Neutra and Kozlowski, 2006; Zhao et al., 2018). Among several mucosal inoculation routes, the nasal and oral delivery are the most reachable and ideal vaccine administration routes (Jabbal-Gill et al., 2012). Orally and nasally delivered potent vaccines are taken up by the gut associated lymphoid tissues (GALT) and nasopharynx-associated lymphoid tissues (NALT), respectively, and elicit local secretory IgA antibodies which are essential for induction of protective humoral immune response (van der Lubben et al., 2001). In addition, mucosal vaccination can target the antigen presenting cells resulting in effective processing of antigens and generation of cell mediated immune response including immunological memory (Mestecky, 1987; Jabbal-Gill et al., 2012). But to achieve effective mucosal vaccination we need a suitable vaccine carrier and/or adjuvant.

**FIGURE 1:**
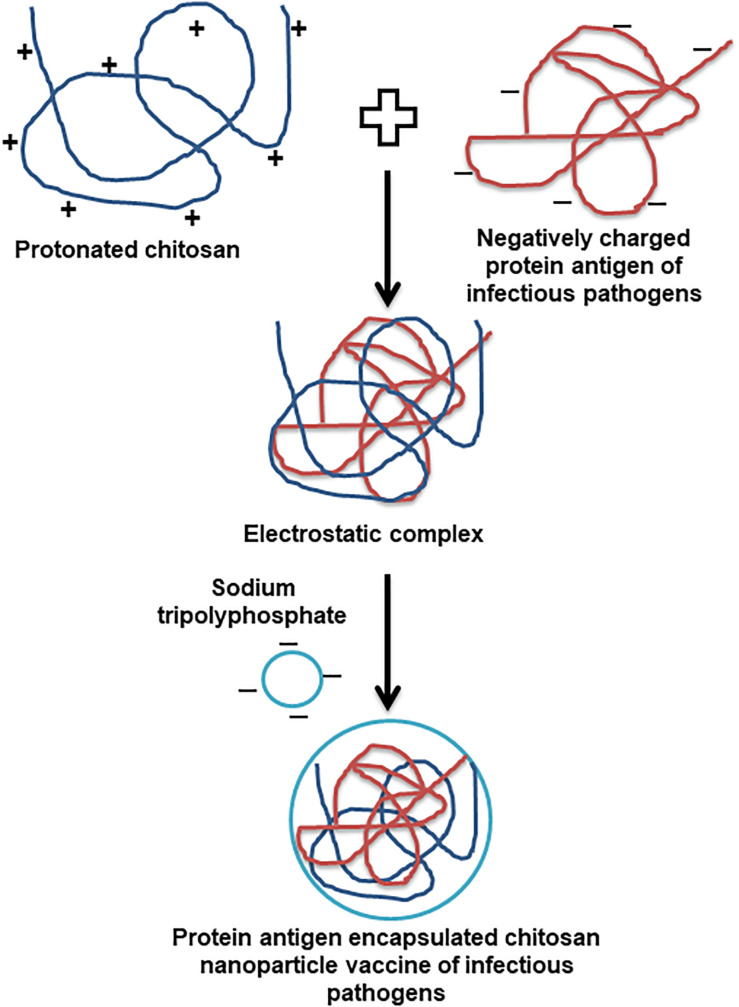
Schematic representation of the preparation of infectious pathogen antigen encapsulated chitosan nanoparticle vaccine.

Owing to the bioavailability, biodegradation, non-toxic, easy to scale up and strong immunostimulatory ability, carbohydrate polysaccharides are the appropriate carriers for developing effective vaccines (Malik et al., 2018). Among diverse polysaccharide polymers, the chitin is the world’s second most abundant natural biopolymer, extracted mainly from the crustacean and insect shells. The chitosan is derived from different degrees of deacetylation of chitin (Bowman and Leong, 2006). The cationic chitosan is a linear copolymer of ß1-4 linked monomers of D-glucosamine and N-acetyl-D-glucosamine (Jabbal-Gill et al., 2012; Samal et al., 2012). The physicochemical and biological properties of chitosan are solely determined by the degree of deacetylation and molecular weight (Jabbal-Gill et al., 2012). Due to primary amino group pKa of chitosan is around 6.5, and it is naturally insoluble in water and soluble in mild acidic condition (Sogias et al., 2008). The soluble and insoluble transition of chitosan happens in pH range of 6 to 6.5, which is appropriate for a range of biological applications, but it possess compatibility issues when antigens are soluble and stable only in natural pH (Dash et al., 2011; Jabbal-Gill et al., 2012). Thus, a lot of structural modifications have been carried out with the primary amine group of chitosan to make it soluble in water without compromising its unique biological applications. The major modifications of chitosan include quaternisation by introducing N-2-hydroxypropyl trimethyl and N,O carboxymethyl (Verheul et al., 2008). Usually chitosan nanoparticles (CS NPs), and vaccine antigen encapsulated CS NPs are prepared by an ionic gelation method using sodium tripolyphosphate (TPP) as a precipitating agent (van der Lubben et al., 2001; Renu et al., 2020c). Generally, for the vaccine preparation pH of transparent chitosan polymer solution is adjusted below 6.5 (4.3 to 5.5) to protonate the chitosan. The encapsulating antigen dissolved above has the isoelectric point (mostly by using pH 7.4 buffer) to make them negatively charged. Due to the natural electrostatic interaction chitosan forms a complex with antigen, and the antigen encapsulated CS NPs vaccine is obtained upon addition of TPP (Figure 1).

The commonly used synthetic and natural mucoadhesive polymers are Cellulose derivatives, Poly (vinyl alcohol), Polyanhydride, Sodium alginate, Starch, and Chitosan. Among them chitosan is widely used to target mucosal sites by oral, ocular, nasal, implant, parenteral, and transdermal administration due to its unique properties such as positive surface charge and flexibility to do modification and conjugation with other polymers (Chaturvedi et al., 2011; Sosnik et al., 2014). The CS NPs have been considered as a novel vaccine delivery vehicle and a potential mucosal adjuvant (Malik et al., 2018), because of (i) its strong surface positive charge facilitating the electrostatic interaction with negatively charged sialic acid in mucus (Illum et al., 2001), leading to increased mucosal absorption of antigen (Dyer et al., 2002); (ii) its capability to open the tight cells junction and promoting permeability of antigens into cells (Kammona and Kiparissides, 2012); and possess (iii) biodegradability, biocompatibility, non-reactogenicity, low cost of production, and immunomodulation ability (Li and Wang, 2015; Zhao et al., 2017). However, chitosan soluble in the acidic environment, thus its use to deliver an acidic pH sensitive antigen is limited. Generally, chitosan forms a polydisperse macroparticle, hence appropriate optimization in the preparation method is required to achieve a desired size and surface charge of the vaccine formulation.

**FIGURE 2:**
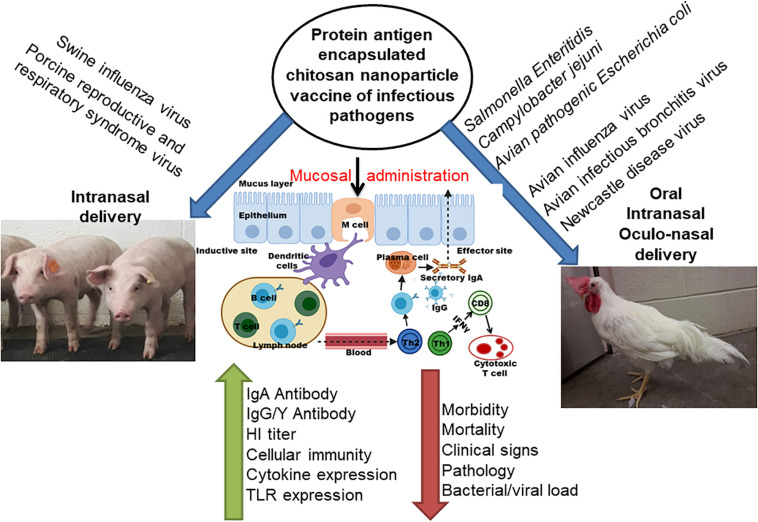
Schematic representation of chitosan based nanoparticle used for delivering bacterial and viral vaccine to mucosal sites of poultry and pigs, and the vaccine induced mucosal immune responses.

**FIGURE 3:**
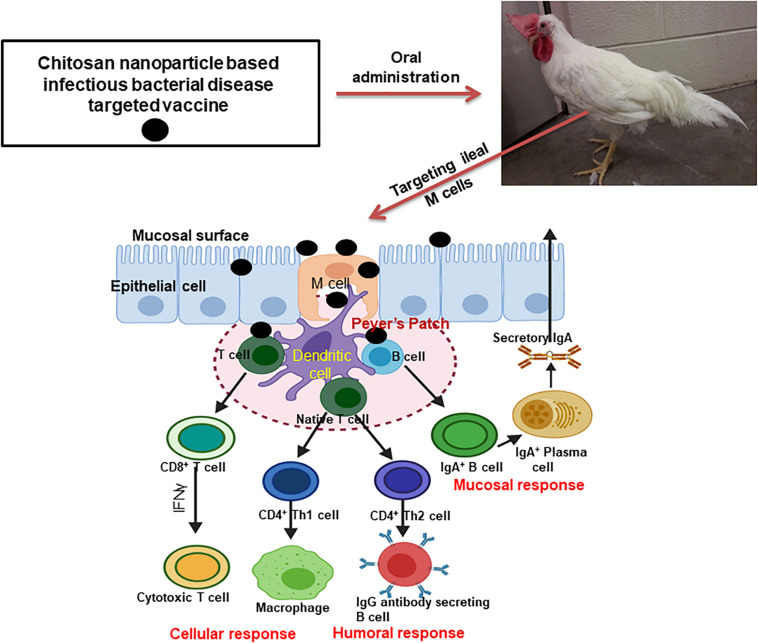
Mechanism of induction of humoral, mucosal and cellular immune responses by oral delivered chitosan nanoparticle based bacterial infectious disease pathogen vaccine in poultry.

**FIGURE 4:**
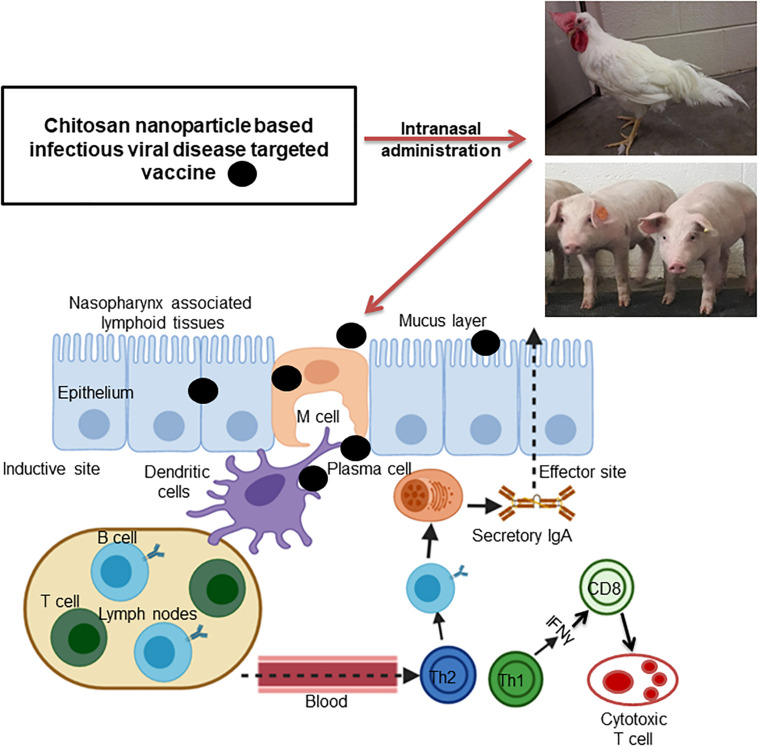
Schematic illustration of intranasally administered chitosan nanoparticle based viral infectious disease pathogen vaccine triggered mucosal and cellular immune responses in the inductive and effector sites of poultry and pigs.

Mucosal immune system consists of mucosa-associated lymphoid tissues (MALT), present along the mucosa of the respiratory tract and gastrointestinal tract as NALT and GALT (Fujkuyama et al., 2012). The orally delivered CS NPs based vaccines overcome the problem of antigen degradation in the gastrointestinal tract and readily up taken into GALT Peyer’s patches (PPs) microfold (M) cells (van der Lubben et al., 2001). Similarly the nasal delivered CS NPs based vaccines prevent rapid mucociliary clearance (Bernocchi et al., 2017) and reach NALT which contain lymphoid follicles (B-cells), inter-follicular (T-cell areas) cells, macrophages and dendritic cells (DCs; Illum et al., 2001). Generally, mucosal delivered CS NPs based vaccines are taken up by M cells in the MALT, transported to sites rich in DCs, macrophages, and B cells resulting in uptake and processing and presentation of antigen, leading to activation of antigen specific CD4^+^ T helper cells which interact with B cells to generate IgA committed cells (IgA^+^ B cells). The IgA^+^ B cells migrate to effector sites, differentiate into IgA producing plasma cells and secrete IgA, leading to induction of mucosal immune response (Fujkuyama et al., 2012; Zaman et al., 2013; Kaur et al., 2018; Jin et al., 2019; Thakur and Foged, 2020; Figures 3, 4). While activation of CD8^+^ T cells and CD4^+^ Th1 cells triggers the generation of cytotoxic T lymphocytes, resulting in stimulation of cellular immune response (Singh et al., 2018; Figures 3, 4). Hence from last one decade CS NPs have been tested widely to deliver vaccines against various bacterial and viral infectious diseases to the mucosal area of native animal hosts [for example, poultry is a native host for *Salmonella* and avian influenza virus, and pigs for swine influenza virus (SIV); Dhakal et al., 2018; Renu et al., 2020c]. In this review, we enlightened up to date progress made on CS NPs based mucosal vaccines for various bacterial and viral infectious diseases of poultry and pigs (Figure 2). We also discussed the CS NPs based vaccines induced immune response and correlation to their protection against challenge infection.

## CS NPs Based Infectious Disease Vaccine Delivered to Mucosal Sites of Poultry

From the last few years poultry is the most consumed meat worldwide. Due to increasing global population, it is expected that major protein source of poultry meat consumption will be double in near future, with 40% increase in consumption of eggs (Astill et al., 2018). Poultry are highly prone for several diseases, and due to its high demand it is extremely challenging to limit the food safety concerns in humans (Liverani et al., 2013). The contamination of meat could happen from many sources and the major once are environmental and human intervention while handling animals (Sofos, 2008). The food producing animals including poultry are the key reservoirs for major foodborne pathogens such as *Salmonella, Campylobacter*, toxin-producing strains of *Escherichia coli* and Listeria monocytogenes, and zoonotic transmission of the latter even cause death in humans (Heredia and Garcia, 2018). Foodborne pathogens induces 1.5 billion loss through causing diarrheas in less than 3 year old children and more than 3 million deaths per year (Heredia and Garcia, 2018). Similarly, viral pathogens also induce severe economic crisis in poultry industry worldwide. The major poultry viral pathogens are avian influenza, infectious bronchitis, infectious bursal disease and Newcastle disease viruses (Brown Jordan et al., 2018). Some avian influenza viruses are threat to humans with a potential to cause pandemic (Astill et al., 2018). Vaccination is the effective way to control poultry infectious diseases as well as prevent zoonotic transmission to humans. Thus, until now CS NPs based vaccines have been used to deliver antigens of *Salmonella Enteritidis*, *Campylobacter jejuni*, avian pathogenic *E. coli*, avian influenza virus, infectious bronchitis virus (IBV) and Newcastle disease virus (NDV) to mucosal sites in poultry (Table 1).

**TABLE 1:** Chitosan and modified chitosan nanoparticle-based vaccines: physicochemical properties, immune responses, and efficacy induced by vaccines administered to mucosal sites to protect against bacterial and viral infectious pathogens of poultry.

Vaccine carrier	Physicochemical properties of chitosan NPs based vaccine	Target pathogens	Encapsulated vaccine antigens	Route of delivery; antigen amount; number of vaccine doses	Target animal	Immune correlates and pathogen clearance	References
Chitosan NPs surface tagged with *Salmonella Enteritidis* flagellin protein	Low molecular weight chitosan Size: 517 nm Zeta potential: — Shape: spherical	*Salmonella Enteritidis*	Outer membrane proteins and flagellin protein	Oral; 100 μg; 3	Layer chickens	Enhanced mucosal IgA antibody, cellular immune response, TLRs gene expression	Renu et al., 2020c
Chitosan NPs surface tagged with *Salmonella Enteritidis* flagellin protein	Low molecular weight chitosan; 75–85% deacetylated; 50,000–190,000 Da Size: 514 nm Zeta potential: +40 mV Shape: spherical	*Salmonella Enteritidis*	Outer membrane proteins and flagellin protein	Oral; 50, 100 and 500 μg; 3	Layer chickens	Increased TLRs, Th1 and Th2 cytokines mRNA expression, antigen-specific humoral immune response; lower bacterial challenge load in cecum	Renu et al., 2020b
Chitosan NPs surface tagged with *Salmonella Enteritidis* flagellin protein	Low molecular weight chitosan Size: — Zeta potential: — Shape: —	*Salmonella Enteritidis*	Outer membrane proteins and flagellin protein	Oral; 500, 1000 and 2000 μg; 2	Broiler chickens	Induced cross-reactive IgG and mucosal IgA antibodies, cytokine gene expression; lower heterologous challenge bacterial load in liver and spleen	Acevedo-Villanueva et al., 2020
Chitosan NPs surface tagged with *Salmonella Enteritidis* flagellin protein	Low molecular weight chitosan Size: 300 nm Zeta potential: +40 mV Shape: spherical	*Salmonella Enteritidis*	Outer membrane proteins and flagellin protein	Oral; 12.5 and 50 μg; 1 and 2	White Cornish Cross broilers	Increased innate immune response and antigen specific lymphocytes proliferation; lower antigen specific IgA and IgG antibody response	Han et al., 2020c
Chitosan NPs surface tagged with *Salmonella Enteritidis* flagellin protein	Low molecular weight chitosan Size: — Zeta potential: — Shape: —	*Salmonella Enteritidis*	Outer membrane proteins and flagellin protein	Oral; 10 and 50 μg; 1 to 3	Cornish Cross breed broilers	Robust pre and post-challenge IgG and IgA antibody, cell mediated immune response, TLRs gene expression; reduced challenge bacterial load in the cecum	Han et al., 2020a
Mannose conjugated chitosan NPs surface tagged with *Salmonella Enteritidis* flagellin protein	Low molecular weight chitosan Size: — Zeta potential: — Shape: —	*Salmonella Enteritidis*	Outer membrane proteins and flagellin protein	Oral; 10 μg; 2	Cornish Cross breed broilers	Enhanced cell mediated immune response, TLRs and balanced Th1 and Th2 cytokine gene expression; reduced *Salmonella* challenge load in the cecum	Han et al., 2020b
Chitosan NPs	Size: 167 nm Zeta potential: +20.1 mV Shape: spherical	*Campylobacter jejuni*	Recombinant hemolysin co-regulated protein	Oral; 50 μg; 3	Vencobb chicks	Increased secretory IgA and systemic IgY antibody, cytokine gene expression; enhanced bacterial clearance in cecum	Singh et al., 2019
Chitosan NPs	85% deacetylated; 400 kD Size: 80–100 nm Zeta potential: — Shape: spherical	*Campylobacter jejuni*	flaA gene	Intranasal; 150 μg; 3	White Leghorn chickens	Increased serum IgG and intestinal mucosal IgA antibody; reduced bacterial shedding by 2–3 log_10_ in large intestine and cecum	Huang et al., 2010
Chitosan NPs	Medium molecular weight chitosan; 75–85% deacetylated Size: — Zeta potential: +19.9 mV Shape: spherical	Avian pathogenic *Escherichia coli*	ΦKAZ14 bacteriophage	Oral; 10^7^ PFU/mL; 1	Broiler chicks	Improved body weight; decreased mortality; decreased bacterial colonization in intestines; reduced fecal shedding; increased protection rate	Kaikabo et al., 2017
Chitosan NPs surface tagged with HA2 and M2e influenza proteins	Low molecular weight chitosan Size: 100–800 nm Zeta potential: — Shape: spherical	Avian influenza virus	HA2 and M2e mRNAs	Intranasal; 4 μg; 2	Layer chickens	Increased IgG and mucosal IgA antibody, virus neutralization titers, cell-mediated immune response; lower lung pathology; reduced homologous and heterologous challenge virus titers in cloacal swab	Hajam et al., 2020
Chitosan	85% deacetylated Size: — Zeta potential: — Shape: —	Avian influenza virus	Inactivated split influenza virus	Intranasal; 100 HA units; 1 and 2	Layer chickens	Enhanced mucosal IgA and HI antibody; resist against lethal virus challenge in field condition	Worrall et al., 2009
Chitosan NPs	Medium molecular weight chitosan; 75–85% deacetylated Size: 286 nm Zeta potential: +19.9 mV Shape: spherical	Avian infectious bronchitis virus	Inactivated infectious bronchitis virus	Oculo-nasal; 10^8^.^285^ EID_50_ of the virus; 1	SPF chickens	Enhanced mucosal IgA, IFNγ gene expression; no or mild relevant microscopic lesions; lower viral load in trachea and kidney	Lopes et al., 2018
N-2- hydroxypropyl trimethyl ammonium chloride chitosan and N,O-carboxymethyl chitosan NPs	Size: 251.8 and 122.4 nm Zeta potential: 46.6 and 53.2 mV Shape: spherical	Newcastle disease virus and infectious bronchitis virus	Newcastle disease virus and infectious bronchitis virus combined	Intranasal; 10^7^.^4^ and 10^5^.^5^ EID_50_ of the virus; 1	SPF chickens	Increased IgG and IgA antibodytiters, lymphocyte proliferation, cytokines IL-2, IL-4 and IFN-γ than the commercial live vaccine; complete protection	Zhao et al., 2017
N-2-hydroxypropyl trimethyl ammonium chloride chitosan and N,O carboxymethyl chitosan NPs	Size: 309.7 nm Zeta potential: 49.9 mV Shape: spherical	Newcastle disease virus	pVAX I-F(o)	Intranasal; 200 μg; 2	SPF chickens	Higher IgG and secretory IgA antibody; stimulated lymphocyte proliferation; increased IL-2, IL-4, and IFN-γ levels; complete protection	Zhao et al., 2018
N-2-hydroxypropyl trimethyl ammonium chloride chitosan and N,O carboxymethyl chitosan NPs	85% deacetylated Size: 252.2 nm Zeta potential: +41.1 mV Shape: spherical	Newcastle disease virus	Attenuated live Newcastle disease virus	Oral or intranasal; 10^7^.^5^ EID_50_ of the virus; 1	SPF chickens	Induced high titers of serum antibody; promoted lymphocyte proliferation; higher levels of serum IL-2, IL-4 and IFN- γ; no clinical signs and mortality	Jin et al., 2017
N-2-hydroxypropyl trimethyl ammonium chloride chitosan NPs	85% deacetylated; 71.3 kDa Size: 303.8 nm Zeta potential: 45.7 mV Shape: spherical	Newcastle disease virus	Attenuated live Newcastle disease virus	Oral or intranasal; 10^7^.^12^ EID_50_ of the virus; 1	SPF chickens	Induced robust cellular, humoral and mucosal immune response; absence of pathological changes; complete protection than the commercial attenuated live vaccine	Zhao et al., 2016
O-2’ hydroxypropyl trimethyl ammonium chloride chitosan NPs	85% deacetylated Size: 303.5 nm Zeta potential: +46.3 mV Shape: spherical	Newcastle disease virus	Attenuated live Newcastle disease virus	Oral or intranasal; —; 1	SPF chickens	Robust cellular, humoral and mucosal immune response; absence of histopathological changes compared to a commercial attenuated live vaccine	Dai et al., 2015
Chitosan NPs	80% deacetylated; 71.3 kDa Size: 199.5 nm Zeta potential: +12.1 mV Shape: spherical	Newcastle disease virus	F gene plasmid DNA	Intranasal; 200 μg; 2	SPF chickens	Increased IgA and IgG antibodies, lymphocyte proliferation response; absence of clinical symptoms and mortality	Zhao et al., 2014
Chitosan NPs	80% deacetylated; 71.3 kDa Size: 371.1 nm Zeta potential: +2.8 mV Shape: spherical	Newcastle disease virus	Lentogenic live Newcastle disease virus	Oral or intranasal; —; 1	SPF chickens	Enhanced lymphocyte proliferation, serum HI and intestinal mucus IgA antibody titers; absence of clinical signs and mortality	Zhao et al., 2012
Chitosan	70–95% deacetylated Size: — Zeta potential: — Shape: —	Newcastle disease virus	Live Newcastle disease virus	Oculo-nasal; 10^6^ EID_50_ of the virus; 1	SPF white Leghorn chickens	Enhanced cell mediated immune response; no effect on systemic and mucosal antibody mediated immune response	Rauw et al., 2010a
Chitosan	—	Newcastle disease virus	Live Newcastle disease virus	Oculo-nasal; 10^6^ EID_50_ of the virus; 1	Isa Brown layer chickens	Provides protection against mortality and morbidity; reduced virus shedding; higher cellular and mucosal antibody mediated immune response	Rauw et al., 2010b

*NPs, nanoparticles; PFU, plaque forming units; HA, hemagglutinin; EID_50_, 50% egg infective dose; HI, hemagglutination inhibition; TLRs, toll-like receptors; Th1, T helper type 1; Th2, T helper type 2; IL, Interleukin; and IFNγ, interferon gamma.*

### CS NPs Based Vaccines for Bacterial Infectious Disease

In this chapter, we summarized studies so far reported using CS NPs and its derivatives based bacterial infectious pathogens vaccines administerd to mucosal sites, and induced immune response and efficacy in poultry. The mechanism of induction of immune response by orally administered CS NPs based vaccine is emphasized in Figure 3.

*Salmonella* is a rod-shaped, Gram-negative bacterium of Enterobacteriaceae family. *Salmonella* enterica serovar *Enteritidis* colonize intestines in poultry without causing disease. *S. Enteritidis* (*S. Enteritidis*) is a major economic threat to poultry industry causing food-borne illness in humans (Khan et al., 2003). In humans, each year *Salmonella* infection is responsible for around 40,000 cases and 400 deaths in the United States (Fabrega and Vila, 2013). Our group has recently reported the CS NPs biocompatibility, stability at various acidic and alkaline pH conditions, and prepared *S. Enteritidis* flagellin protein surface coated CS NPs to mimic natural bacterium colonization in the ileum of chickens. Through *in vitro* and *in vivo* studies, we demonstrated that surface modified CS NPs was up taken by chicken immune cells and reached ileum lamina propria and PPs sites. The *S. Enteritidis* subunit antigens, outer membrane proteins (OMPs) and flagellin protein entrapped and surface flagellin protein coated CS NPs (nanovaccine) delivered orally in layer chickens induced antigen specific IgA response in cloaca but did not elicit the IgG antibody response in serum. In nanovaccine inoculated *Salmonella* challenged birds observed enhanced anamnestic specific IgA response in cloaca, ileum and bile samples, with increased serum IFN-γ level and antigen specific lymphocyte proliferation responses were observed. *Salmonella* nanovaccine immunization also increased the Toll-like receptor (TLRs)-2 and -4, IFN-γ, TGF-ß, and IL-4 gene expression. Although due to high bacterial challenge dose [1 × 10^9^ colony-forming unit (CFU) per bird] used in that nanovaccine trial we did not detect substantial reduction in the bacterial colonization, but our this first study confirmed that surface flagellin protein coating on CS NPs is necessary to reach the vaccine to ileum immune cells of layer chickens when delivered orally (Renu et al., 2020c). To determine the protective efficacy of nanovaccine, in another study (Renu et al., 2020b), we optimized CS NPs by altering the concentrations of chitosan and TPP ratios used in the vaccine formulation as well as experimental conditions. The optimized CS NPs surface modified with flagellin protein delivered orally in chickens reached PP’s and ileum, and *in vitro* treatment of the nanovaccine in chicken immune cells increased the TLRs 1, 2, 3, 4, 5, 7, 15, and 21, and IFN-γ, IL-2, IL-4, and IL-10 gene expression. In contrast to our previous study, the optimized nanovaccine oral immunization in layer chickens increased the specific IgY antibody level in serum. Delivery of the vaccine through oral gavage, drinking water, and feed increased the antigen specific IgY and IgA antibody response following the optimized dose of *Salmonella* challenge (5 × 10^6^ CFU) infection. Oral gavage and drinking water delivered nanovaccine immunization induced immune response correlated with reduction in *Salmonella* load in cecum (Renu et al., 2020b). Both our study outcomes confirmed that flagellin protein surface conjugation of CS NPs is necessary to improve the efficacy of oral *Salmonella* vaccine in layer chickens; two doses of nanovaccine inoculation is sufficient and the third dose did not further boost the antibody response; and the optimized bacterial challenge dose is crucial to appreciate the vaccine induced protective efficacy. Importantly, nanovaccine can be delivered in drinking water and feed, but needs higher dose of vaccine to elicit immune response and reduce bacterial shedding.

Broiler birds are highly vulnerable to *Salmonella* infection, and the use of killed vaccine to control infection is not recommended as the parenteral injection causes stress, reduce production and meat quality (Han et al., 2020c). Thus, efficacy of needle-free oral deliverable optimized nanovaccine was evaluated in broilers. In our pilot study (Acevedo-Villanueva et al., 2020) experiment 1: following two oral delivered very high doses of nanovaccine (500, 1,000, and 2,000 μg antigen in CS NPs) and *S. Enteritidis* challenge infection detected induction of high levels of nitrite production in macrophages and secretion of antigen specific IgG and IgA antibodies at early time points. The inoculated nanovaccine based on encapsulated antigen concentration expressed various levels of cytokines IL-1β, IL-10, and IL-4 mRNA. In experiment 2: nanovaccine immunization and *S. Enteritidis* or *S. Heidelberg* challenge in birds increased the antigen specific cross-reactive IgG and IgA antibodies at early time point and cleared the *S. Heidelberg* load in liver and spleen, and *S. Enteritidis* burden in cecal content. The nanovaccine induced antibodies and reduced bacterial load was comparable to the commercial live *Salmonella* vaccine (Acevedo-Villanueva et al., 2020).

In another study in broilers, like in layers, the oral delivered nanovaccine targeted ileum and reached lamina propria. However, two doses of nanovaccine (12.5 and 50 μg antigen in CS NPs) inoculation only increased different TLRs mRNA expression and specific splenocytes proliferation, but did not induce high levels of antigen specific antibody response (Han et al., 2020c). Likewise, the oral administration of killed *S. Enteritidis* whole protein antigen encapsulated in CS NPs also failed to induce an adaptive immune response (Han et al., 2020c). Whereas, lower dose (10 μg antigen), vaccination at young age (3rd day), and two or three doses of nanovaccine administration heightened the systemic and mucosal antibody, cell mediated immune response, TLRs gene expression, resulting in reduced *S. Enteritidis* load in the cecum (Han et al., 2020a). Mannose receptor is expressed on dendritic cells and macrophages, targeting them using mannose ligand conjugated nanovaccine elicited enhanced cell mediated immune response, and in without mannose conjugated nanovaccine received birds observed increased mucosal antibody response, while both the nanovaccines formualtions reduced the challenge *Salmonella* load in the intestines comparable to a commercial live vaccine (Han et al., 2020b). Altogether, our broiler experiments revealed that the selection of antigen, vaccine dose, age of birds vaccinated and the type of modifications on CS NPs are important to achieve robust immune response and protective efficacy against *Salmonella*. In our experiments both in layer and broiler chickens, oral inoculation of nanovaccine reduced the bacterial load by inducing mucosal IgA antibody and cytokine gene expression. Further, to widen the breadth of cross-protection in chickens, we need to include multiple *Salmonella* serotype derived antigens and secondary adjuvants in CS NPs vaccine formulation.

*Campylobacter jejuni* (*C. jejuni*) is a helical-shaped, non-spore forming; Gram-negative enteric bacterium belongs to Campylobacteraceae family. It is a most common food borne pathogen globally. Poultry is the natural host for *C. jejuni* and contaminated meat is the main source for human infection (Pielsticker et al., 2012). In the United States, each year around 2.4 million Campylobacter related human illness cases are reported (Sean et al., 1999). Recently, CS NPs was used to deliver recombinant hemolysin co-regulated protein of *C. jejuni* in chickens (Singh et al., 2019), and used molecular techniques to confirm the loaded antigen and found it was intact in the CS NPs, and oral vaccination increased the pre-challenge secretory IgA and systemic IgY antibodies. Vaccination also increased NFkB, IL-1β, IL-8, IL-6, IFN-γ, and IL-17A gene expression, and reduced the challenge *C. jejuni* load in cecum. Although subcutaneous injection of antigen with traditional incomplete Freund’s adjuvant induces superior antibody response, the bacterial clearance was lower than oral inoculation of CS NPs based vaccine (Singh et al., 2019). Interestingly, major structural protein FlaA gene based CS NPs-DNA vaccine inoculated three times intranasally in chickens elicited the serum IgG and mucosal IgA antibodies, and CD4^+^/CD8^+^ T cells ratio and rendered 2–3 log_10_ reduction in bacterial load (Huang et al., 2010). Moreover, in chickens downward trend in positive cases of *C. jejuni* over a period and remain not detectable after 21 days. Notably, the chitosan based DNA complex vaccine induces lower levels of immune response and weaker clearance of pathogen, suggesting that delivery of antigen entrapped in CS NPs vaccine is better than just mixing with chitosan (Huang et al., 2010).

*Escherichia coli* is a rod-shaped, Gram-negative enteric bacterium, belongs to Enterobacteriaceae family and it is a commensal. Avian pathogenic *E. coli* (APEC) stay in the intestines and causes disease in other organs and systemically (Kaikabo et al., 2017). The APEC causes Colibacillosis and septicemia, induced high mortality in poultry leading to severe economic loss worldwide (Delicato et al., 2003; Ewers et al., 2004). A single dose of bacteriophage encapsulated CS NPs delivered orally in broilers increased body weight, decreased mortality, reduced fecal shedding of APEC and viable bacterial counts in major vital organs with increased protection (Kaikabo et al., 2017). This study recommends that bacteriophage-based CS NPs treatment is beneficial for increasing the body weight gain and in control of other enteric pathogenic bacterium in poultry. Overall, all these studies suggest that CS NPs could be used efficiently to deliver vaccines loaded with bacterial pathogens to mucosal sites in poultry. Mostly, the delivered vaccines increased secretory IgA and IgG antibody, in some cases cellular immune response, thereby reduced pathogenic bacterial shedding and offered protection. However, the number of studies conducted so far are limited and more research is required to establish whether CS NPs is a suitable vaccine carrier for bacterial infections in poultry.

### CS NPs Based Vaccines for Viral Infectious Disease

Under this section, we highlighted the studies conducted using CS NPs and its derivatives to deliver viral infectious disease targeting vaccines administered to mucosal sites which triggered immune response and protection in poultry. A schematic illustration of intranasally administered nanovaccine eliciting mucosal and cell mediated immune responses are shown in Figure 4.

Influenza virus belong to the family Orthomyxoviridae, and there are A, B, and C types of virus present based on its antigenic differences (Horimoto and Kawaoka, 2001). All the identified avian influenza virus (AIV) belongs to type A and designated either as highly pathogenic avian influenza (HPAI) or low pathogenic avian influenza (LPAI; Hajam et al., 2020). The AIV infections cause significant economic losses in poultry industry worldwide (França and Brown, 2014). Globally, wild aquatic birds are the natural reservoir for AIV and infects domestic poultry, other birds, animal species and even humans (Yiu Lai et al., 2013). Recent study has shown that CS NPs is efficiently taken up by chicken macrophages, and its intranasal treatment in layer chicken adhered to nasal mucosa (Hajam et al., 2020). Further, the conserved HA2 and M2e influenza proteins surface coated and its mRNA encapsulated CS NPs vaccine delivered intranasal in layer chickens elicited systemic IgG and mucosal secretory IgA antibodies response, cross-reactive serum virus neutralization antibody titers and T-cell response (Hajam et al., 2020). Although intranasal vaccination induced immune responses correlated with reduced lung pathology, cross-protective efficiency was checked in the cloaca but not in lungs. This study recommended that delivery of antigen both in mRNA and protein form using CS NPs is more efficient compared to antigens delivered as such in terms of inducing immune response and protection against AIV (Hajam et al., 2020). In another study, inactivated three field AIV strains mixed with the bacterial adjuvant *Clostridium perfringens* and chitosan (not in CS NPs platform) delivered intranasal in chickens induced anamnestic haemagglutination inhibition (HI) titers and mucosal IgA response. In the field studies, CS NPs vaccination efficiently controlled AIV outbreaks in chicken with high HI antibody titer, rescued the loss in egg production without infection, while the commercial intramuscular inactivated AIV vaccine failed to provide protection. This study suggests that combination of intranasal vaccine with commercial intramuscular vaccine is more comprehensive as a prophylaxis against the disease, and they used chitosan instead of CS NPs (Worrall et al., 2009). Over all, this study recommends that using appropriate antigen, secondary adjuvants, with chitosan, and proper route of immunization could induce early mucosal sIgA and subsequent humoral IgG antibodies and prevent AIV outbreaks in the field (Worrall et al., 2009).

Infectious Bronchitis belongs to a Coronaviridae family. It is a single stranded RNA virus. Chickens are reservoirs for the contagious IBV affecting the upper respiratory tract, female reproductive tract, and causes nephritis in all age group birds. In chickens, IBV typically causes 100% morbidity, less than 50% mortality, stunted growth, poor carcass weight, and decreased egg quality and production (Mark, 2012; Bande et al., 2016). A study has shown that single dose of inactivated IBV encapsulated in CS NPs administered by oculo-nasal route in chickens reduced ciliostasis and viral RNA copies in trachea with undetectable copies in kidney. This vaccine triggered early IFN-γ gene expression, increased IgA level, and reduced approximately 70% pathology in trachea and kidney of chickens. Although commercial live vaccine prime and CS NPs vaccine boost increased the IgG response and IFN-γ expression, reduced IBVs copy numbers was not greater than only CS NPs based vaccine received birds (Lopes et al., 2018). Suggesting that an appropriate vaccine formulation, dose and route of delivery can offer better protection against IBV in chickens.

Newcastle disease is caused by a negative-sense, single-stranded RNA virus called NDV, and it belongs to the Paramyxoviridae family. The NDV causes high morbidity and mortality as well as drop in egg production in chickens (Brown and Bevins, 2017; Absalón et al., 2019). The last NDV outbreak in the United States resulted in culling of 3.16 million birds (Brown and Bevins, 2017). In a study (Zhao et al., 2017), developed a water soluble chitosan derivative called N-2-hydroxypropyltrimethyl ammonium chloride chitosan and N,O-carboxymethyl chitosan to prepare IBV and NDV individual/combined vaccine. The intranasal vaccination of such chitosan derivative based CS NPs vaccine in chickens elicited antigen specific IgG and mucosal IgA antibodies, lymphocytes proliferation and IFN-γ, IL-2, and IL-4 gene expression. Although compared to commercial attenuated live vaccine, the modified CS NPs based vaccine offered complete protection against both IBV and NDV, but a control unmodified CS NPs based vaccine was not included in that study to reveal the need of such a chitosan modification (Zhao et al., 2017). The same research group in another study modified water soluble CS NPs encapsulated pVAX I-F(o) DNA along with C3d6 molecular adjuvant in the vaccine. Like the previous study (Zhao et al., 2017), intranasally delivered CS NPs based vaccine in chickens induced higher IgG and secretory IgA antibodies, lymphocyte proliferation, IL-2, IL-4, and IFN-γ level, and produced complete protection against NDV. The intranasal delivered vaccine induced higher immune response compared to intramuscular immunization (Zhao et al., 2018). This concludes that CS NPs based vaccine delivered intranasally not only increased the secretory IgA but also systemic IgG antibody and cellular immune response which was correlated with protection against the infection. Since, in this study only DNA and C3d6 molecular adjuvant was not used in control groups for intranasal inoculation, further studies are required to understand the role of C3d6 molecular adjuvant in vaccine formulation (Zhao et al., 2018).

The modified water soluble chitosan was also used to encapsulate attenuated live NDV in CS NPs, and the vaccine was delivered orally and intranasally in chickens which induced higher and long lasting IgG and IgA antibodies and lymphocyte proliferation response than the control commercial live attenuated NDV vaccine (Jin et al., 2017). The CS NPs vaccination increased IL-2, IL-4, and IFN-γ level, and protected chickens completely against NDV without any pathological and histopathological changes. Even 3 months stored CS NPs vaccine, delivered intranasally provided complete protection. This study concludes that CS NPs can also be used to deliver the live virus as a vaccine, and intranasal inoculation is more efficient compared to oral delivery against NDV infection (Jin et al., 2017). The same research group in another study used only N-2-hydroxypropyl trimethyl ammoniumchloride modified chitosan and not N,O-carboxymethyl chitosan encapsulated attenuated live NDV in CS NPs, and when delivered orally shown high antibodies and cellular immune response compared to intranasal route; however, both oral and intranasal delivered CS NPs vaccine induced absence of any pathological and histopathological changes, and offered complete protection against NDV in chickens, while the commercial live vaccine not did (Zhao et al., 2016). In contrast, using the same vaccine formulation in another study, but with reduced vaccine dose the intranasal delivered CS NPs vaccine induced higher immune response than delivered orally (Dai et al., 2015). The same research group in their earlier two independent experiments used unmodified CS NPs. The F gene plasmid or live NDV encapsulated in unmodified CS NPs delivered oral or intranasal to chickens induced systemic and mucosal antibodies, and humoral and cellular immune response with complete protection against NDV (Zhao et al., 2012, 2014). In conclusion, based on the seven vaccine trials using modified or unmodified CS NPs, appropriate antigen, dose, secondary adjuvant, and route of vaccination helps to achieve a broader and long lasting protective immune response in chickens against NDV.

When chitosan was co-administered with live NDV by oculo-nasal route observed induction of cellular but not humoral immune response in chickens (Rauw et al., 2010a). Whereas in layer chickens vaccinated using herpesvirus recombinant fusion gene of NDV followed by chitosan co-administered with live NDV by oculo-nasal route observed induced both antibody and cell mediated immune response and offered protection against early and late NDV challenge infection (Rauw et al., 2010b). Overall, all these studies recommended that CS NPs based mucosal vaccine delivery platform can offer better protection against viral pathogens in chickens through inducing higher mucosal and systemic antibodies and/or cellular immune response when used with appropriate antigen, dose, secondary adjuvant, and route of delivery.

## CS NPs Based Viral Infectious Disease Vaccine Delivered Intranasally to Pigs

Pork is the most consumed meat worldwide. Due to high demand for animal-based protein consumption, the pork production accounts for one-fourth of the total protein consumed. As the pork production has increased over a period of several years, food security concerns also increased (VanderWaal and Deen, 2018). Pigs are prone for many zoonotic emerging pathogens and pose threat for rapid transmission to humans (Morgan and Prakash, 2006). Viral infections cause harm to pig health and responsible for significant economic burden to the pork industry through morbidity, mortality, loss of production as well as cost involved in control and prevention of diseases (Dhakal and Renukaradhya, 2019). For example, classical swine fever, foot and mouth disease, and African swine fever have caused huge economic impact to swine industry worldwide (Beltran-Alcrudo et al., 2019). The most common viral pathogens of pigs are porcine reproductive and respiratory syndrome (PRRS) virus, SIV, porcine epidemic diarrhea virus, porcine circovirus-2, and foot and mouth disease virus (Dhakal and Renukaradhya, 2019). In this section, we discussed the studies so far used CS NPs to deliver viral infectious pathogens antigen intranasally, and the mechanism of vaccine induced immune response (Figure 4) and efficacy in pigs (Table 2).

**TABLE 2:** Chitosan nanoparticle-based vaccines: physicochemical properties, immune responses, and efficacy induced by vaccines administered by intranasal route to protect against viral infectious diseases of pigs.

Vaccine carrier	Chitosan NPs based vaccine: physicochemical properties	Target pathogens	Encapsulated antigens	Route of delivery; antigen amount; number of vaccine doses	Target animal	Immune correlates and pathogen clearance	References
Chitosan NPs	Low molecular weight chitosan Size: 141 nm Zeta potential: +30.7 mV Shape: -	Swine influenza virus	Whole inactivated influenza virus	Intranasal; 10^7^ TCID_50_ of the virus; 2	Pigs	Induced cross-reactive serum HI titers, cell mediated immune response, cytokine gene expression; partially reduced heterologous lung pathology and virus load in nasal passage comparable to the commercial vaccine	Renu et al., 2020a
Chitosan NPs	Low molecular weight chitosan Size: 571.7 nm Zeta potential: +1.69 mV Shape: spherical	Swine influenza virus	Whole inactivated influenza virus	Intranasal; 10^7^ TCID_50_ of the virus; 2	Pigs	Enhanced mucosal secretory IgA and serum IgG antibody; reduced macroscopic and microscopic pulmonary lesions; reduced virus titers in nasal swab and BAL fluid	Dhakal et al., 2018
Chitosan and alginate NPs loaded with bee venom	—	Porcine reproductive and respiratory syndrome virus	None	Intranasal; -; 1 to 3	Pigs	Enhanced Th1-related and reduced Treg-specific immune response; increased IFN-γ secreting T cells; decreased body temperature; reduced lung lesions; lower viral load in serum and tissues	Lee et al., 2018

*NPs, nanoparticles; TCID_50_, 50% cell culture infectious dose; HI, hemagglutination inhibition; Th1, T helper type 1; IFNγ, interferon gamma; and BAL, bronchoalveolar lavage fluid.*

The swine influenza in pigs is caused by type A influenza virus belongs to Orthomyxoviridae family. SIV infections in pig causes significant economic losses to the pork industry, specifically when combined with other respiratory pathogens (Borkenhagen et al., 2019). SIV infection leads to acute febrile respiratory disease in pigs of all ages (Janke, 2013). Pig serves as a mixing vessel for reassortment of avian and human influenza viruses, for example the triple reassortant 2009 pandemic flu virus (Mastin et al., 2011; Borkenhagen et al., 2019; Loubet et al., 2019). To mitigate SIV in pigs our group has been using inactivated SIV as an antigen to encapsulate in CS NPs. *In vitro* studies revealed that CS NPs based vaccine was internalized by pig immune cells and increased the secretion of cytokines IFN-α, TNF-α, IL-1β, IL-12, IL-6, and IL-10 (Dhakal et al., 2018). CS NPs based SIV vaccine delivered intranasally as mist in pigs enhanced the cross-reactive mucosal IgA and IgG antibodies and increased the recall IFN-γ secretion, resulted in reduced heterologous challenge virus titers in the airways; but the vaccine did not substantially augment the innate cytokine, HI titers and cell mediated immune response (Dhakal et al., 2018). To improve the efficacy of CS NPs based SIV vaccine, in a subsequent study the CS NPs formulation was optimized by increasing the monodispersity of particles and surface charge, and encapsulated the inactivated SIV and TLR-3 ligand poly(I:C) separately in NPs. In pigs, co-administered intranasally with CS NPs based SIV and poly(I:C) vaccine observed increased cytokine gene expression, cell mediated immune response, cross-reactive HI titers and reduced lung pathology, associated with reduced heterologous challenge virus load in the airways akin to intramuscular delivered multivalent commercial SIV vaccine (Renu et al., 2020a). But this vaccine formulation failed to increase both systemic and mucosal antibodies response. Overall, our data suggested that poly(I:C) delivered with SIV antigen in CS NPs induced Th1 biased response, and when delivered without poly(I:C) triggered Th2 biased response, however, both helped to reduce heterologous challenge SIV titer in the airways (Dhakal et al., 2018; Renu et al., 2020a). Overall, our studies recommend that selection of appropriate vaccine formulation is important to induce a strong immune response. In our future studies, to improve the cross-protective efficiency of modified CS NPs based SIV vaccine in both maternal antibody positive and negative pigs, we are planning to use split SIV antigen, cocktail of multiple inactivated SIVs administered with or without a secondary adjuvant. In summary, based on our studies, the CS NPs is a suitable vehicle to deliver SIV vaccine intranasally in pigs, but further optimization of CS NPs-SIV vaccine is required to achieve the broader cross-protection.

Porcine reproductive and respiratory syndrome is caused by a single stranded positive-sense RNA virus called PRRS virus (PRRSV), and it belongs to the family Arteriviridae (Lunney et al., 2016). Pig is the only known host for PRRSV infection, and it causes reproductive failure in sows and respiratory disease in weaned and growing pigs with 2 to 100% mortality (Lunney et al., 2016; Montaner-Tarbes et al., 2019). Globally, PRRS causes significant economic impact to swine industry (Neumann et al., 2005; Montaner-Tarbes et al., 2019). In a study, along with chitosan the alginate was used in preparing nanoparticle encapsulated bee venom, delivered intranasally in pigs which leads to enhanced Th1 response and PRRSV specific antibody production in infected pigs, while only the alginate nanoparticle did not induce higher immune response (Lee et al., 2018). 1 week before and 2 weeks after the PRRSV infection, only in high dose of CS NPs containing bee venom induced neutralizing antibodies and IFN-γ secreting T cell response, which reduced fever, lung pathology and viral genome copies in PRRSV infected pigs. Although bee venom is not specific to PRRSV but when delivered with CS NPs induced immune response and correlated with disease protection (Lee et al., 2018). This study demonstrated that bee venom can be used as an adjuvant to viral antigens when delivered in CS NPs to achieve protective immune response in pigs.

## Conclusion and Future Directions

Poultry and pigs are susceptible to many bacterial and viral infections. Experimental vaccine trials conducted in a natural host of disease is more appropriate than in rodent models as they lead to quick translation of technology to make a commercial product. Pig and poultry may serve as an appropriate animal models to study performance of respiratory and enteric vaccines, respectively. Pig anatomy, genetics and lung structure and physiology are more similar to humans, and thus pig is considered as a useful biomedical animal model for human respiratory diseases. The CS NPs vaccine delivery platform has been used efficiently to administer some of the viral and bacterial vaccines to mucosal sites of pigs and poultry, and still there is a scope to evaluate CS NPs platform for many other infectious and zoonotic diseases. The CS NPs vaccine delivery strategy used to mitigate infectious diseases in poultry and pigs showed benefits with induction of robust mucosal secretory IgA antibody and variable levels of systemic antibodies, cytokine production and cell mediated immune response. Further studies are required to improve efficiency of the vaccine through innovative modifications to the CS NPs vaccine technology. This include application of suitable surface modifications in CS NPs, optimizing its size, shape and surface charge depending on the route of delivery. Further, to achieve increased breadth of immunity and balanced Th1-Th2 immune response for superior cross-protection against many viral and bacterial diseases the following parameters are important: (i) type of antigen and its concentration, (ii) secondary adjuvant/s, (iii) age of animal at the time of prime vaccination, (iv) number of doses, and (v) route of vaccination.

## Author Contributions

SR conceived and wrote the manuscript. GR edited and revised the manuscript. Both authors read and agreed the manuscript for publication.

## Conflict of Interest

The authors declare that the research was conducted in the absence of any commercial or financial relationships that could be construed as a potential conflict of interest.
